# Exploring what is important to patients with regards to quality of life after experiencing a lower limb reconstructive procedure: a qualitative evidence synthesis

**DOI:** 10.1186/s12955-021-01795-9

**Published:** 2021-05-31

**Authors:** H. Leggett, A. Scantlebury, A. Byrne, M. Harden, C. Hewitt, G. O’Carroll, H. Sharma, C. McDaid, the PROLLIT study collaborators, Joy Adamson, Kim Cocks, Joel Gagnier, Paul Harwood, David Ferguson, Reggie Hamdy, Nando Ferriera

**Affiliations:** 1 York Trials Unit, The University of York, York, YO10 5DD UK; 2 Centre for Reviews and Dissemination, The University of York, York, YO10 5DD UK; 3 Hull University Teaching Hospitals, Hull, HU3 2JZ UK

**Keywords:** Quality of life, Lower limb reconstruction, Qualitative evidence synthesis, Thematic synthesis, Patient reported outcome measures

## Abstract

**Background:**

Patient reported outcome measures (PROMs) are used to understand the impact of lower limb reconstruction surgery on patients’ quality of life (QOL). Existing measures have not been developed to specifically capture patient experiences amongst adults with lower limb conditions that require reconstruction surgery. This review aimed to synthesise qualitative evidence to identify what is important to patients requiring, undergoing, or following reconstructive surgery for lower limb conditions.

**Methods:**

MEDLINE, Embase, PsychINFO and Cinahl were searched from inception until November 2020. Studies were included if they employed qualitative research methods, involved patients requiring, undergoing or following lower limb reconstruction and explored patients’ experiences of care, treatment, recovery and QOL. Mixed methods studies that did not separately report qualitative findings, mixed population studies that were not separately reported and studies in languages other than English were excluded. Included studies were analysed using thematic synthesis. The Critical Appraisal Skills Programme qualitative studies checklist was used to undertake quality assessment.

**Results:**

Nine studies met the inclusion criteria. The thematic synthesis identified two overarching themes: (1) areas of living key to QOL for lower limb reconstruction patients and (2) moving towards a new normal. The way in which lower limb reconstruction affects an individual’s QOL and their recovery is complex and is influenced by a range of inter-related factors, which will affect patients to varying degrees depending on their individual circumstances. We identified these factors as: pain, daily functioning and lifestyle, identity, income, emotional wellbeing, support, the ability to adapt and adjust and the ability to move forwards.

**Conclusions:**

The way patients’ QOL is affected after a lower limb reconstruction is complex, may change over time and is strongly linked to their recovery. These findings will aid us in developing a conceptual framework which identifies the outcomes important to patients and those that should be included in a PROM. Further research is then required to establish whether the range of factors we identified are captured by existing PROMs. Depending on the outcome of this work, a new PROM for patients following lower limb reconstruction may be required.

**Supplementary Information:**

The online version contains supplementary material available at 10.1186/s12955-021-01795-9.

## Introduction

A range of lower limb conditions can result in the need for reconstructive surgery including congenital abnormalities, neoplasia (development of tumours), trauma, infection, arthritis, or paralysis [1]. With recent improvements in reconstructive surgery techniques, the salvage rate of lower limb conditions has increased. However, returning the patient to acceptable levels of physical functioning and Quality of Life (QOL) may take many months or even years [2]. This can involve multiple surgical procedures, prolonged hospital stays, and a long rehabilitation process which can be both physically and psychologically burdensome on the patient [3].

To gather an understanding of the impact of lower limb injuries/conditions and surgical reconstruction on a patient’s QOL, healthcare professionals and researchers utilise patient-reported outcome measures (PROMs). These measures aim to understand from the patients’ perspective, the overall effect of the injury or condition, treatment, rehabilitation and recovery on daily life and well-being, including the wider patient experience and their physical, social and psychological functioning [4]. PROMs can therefore be used to guide healthcare professionals’ understanding of patients’ experiences of major lower limb treatment and recovery.

Currently, a range of condition-specific and generic PROMs are commonly used for adult lower limb conditions including the Olerud-Molander Ankle Score (OMAS) [5] and the Disability Rating Index (DRI) [6] which assess musculoskeletal function and generic measures such as the Sickness Impact Profile (SIP) [7], the Short-Form-36 (SF-36) [8], and the Nottingham Health Profile (NHP) [9]. The Patient-Reported Outcomes Measurement Information system (PROMIS) was initiated by a multi-centred cooperative group to build and validate common measures of key symptoms and health concepts amongst a range of chronic conditions [10]. Rothrock et al. [11] validated the PROMIS Physical Function 8a Short Form in patients with lower limb orthopaedic trauma and demonstrated excellent internal consistency reliability and convergent validity. However, QOL assessments were not included in this measure and the sample population was limited to patients with an isolated lower limb fracture. A systematic review of PROMs for patients undergoing limb circular frame fixation concluded that there is a lack of PROMs that are truly representative of health outcomes for patients with lower limb conditions requiring reconstructive surgery [12]. Research is currently ongoing to address the gap for paediatric patients by Chhina et al. [13] who are developing a PROM specifically for children and adolescents with lower limb deformities. Since we started our systematic review we have also become aware of work currently underway at a United States research centre to develop a PROM for adult patients with severe lower limb extremity injuries requiring reconstruction surgery and amputation in adult patients [14, 15].

To our knowledge, there are no current assessment tools that are developed specifically for this group of patients; as such, current tools may not accurately capture important adult patient experiences amongst populations with lower limb conditions requiring reconstruction surgery. This review aims to review the existing research evidence on what is important to patients requiring, undergoing, or following reconstructive surgery for lower limb conditions. We did not want to impose pre-defined boundaries of what should be considered as important to patients and so we deliberately chose to explore QOL in the broadest sense and included aspects such as: physical functioning, lifestyle, emotional well-being and the way patients are treated and cared for in our review.

**Fig. 1:**
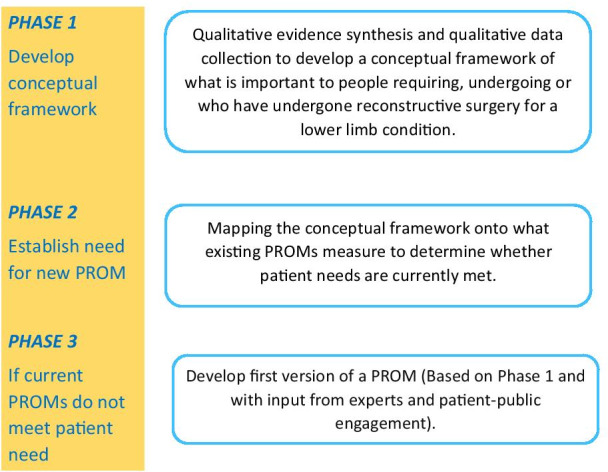
Diagram of the phases of the PROLLIT study [16]

The review is being conducted as part of the wider “PROLLIT” (Patient Reported Outcome Measure for Lower Limb Reconstruction) study [16]. The findings of this review will be used to determine whether existing measures are ‘fit for purpose’ in terms of whether they address areas relevant to this specific patient group or whether a new PROM for lower limb reconstruction surgery patients is required (Fig. 1). As such, the findings from this review will inform topic guides for a qualitative study with health professionals and patients to explore the identified areas of importance to patients. The findings of both this review and the primary qualitative study will be used to develop a conceptual framework to identify and map what is important to patients during or after undergoing reconstructive surgery for a lower limb condition.

## Methods

The methods for Qualitative Evidence Synthesis (QES) published by the Cochrane Qualitative and Implementation Methods Group [17] provided the methodological framework for the design and implementation of this review. The review was prospectively registered on the PROSPERO database (CRD42019139587).

### Search strategy for the identification of relevant studies

A search strategy was developed in Ovid MEDLINE by an Information Specialist (MH) in conjunction with the review team. The strategy consisted of a set of terms for the population of interest combined using the Boolean operator AND with a set of terms covering qualitative research methods. Search terms relating to qualitative methods included thesaurus terms, as well as specific and broad free-text terms [18]. Retrieval was limited to publications in English. The MEDLINE strategy was adapted for use in all other databases. The following databases were searched: MEDLINE (Including: Epub Ahead of Print, In-Process & Other Non-Indexed Citations, MEDLINE Daily and MEDLINE, via Ovid), Embase (Ovid), PsycINFO (Ovid), and CINAHL Complete (Ebsco). Searches were conducted from inception to August 2019 and were limited to publications in English. Updated searches were conducted in November 2020. All search strategies can be found in Additional file 1. A manual search of the reference list of included studies was also undertaken to identify other studies meeting the inclusion criteria.

### Inclusion and exclusion criteria

The SPIDER [19] framework was used to develop the inclusion and exclusion criteria (Table 1).

**Table 1:** Eligibility criteria in line with the SPIDER framework

	Inclusion	Exclusion
Sample	Adult patients (aged 16+) requiring, undergoing or who have undergone reconstructive surgery for a lower limb condition (leg, ankle or foot). Conditions may include: a fracture fixation which becomes infected; non-union (a fracture which does not heal); malunion/deformity (a fracture which has healed in an incorrect position); any acquired or congenital condition leading to bone deformity, leg length discrepancy or bone loss, congenital lower limb deformities, joint contracture; lower limb injuries where further limb reconstruction is required; poly-trauma patients (as long as one of the above criteria were met) Patients were included at any time point after injury or condition onset Studies with a mix of amputee and reconstruction patients were included if the results for both groups of patients could be separated	Those under the age of 16 Patients who have undergone a lower limb amputation
Phenomenon of interest	QOL, including (but not limited to) social interactions, employment, perceived health and QOL after condition onset/injury and throughout recovery This inclusion was kept broad since defining QOL as a single entity is challenging due to individual perceptions as to what constitutes quality living [20]	–
Design	Studies which used established qualitative methods such as interviews or focus groups and used established qualitative analytical approaches (e.g. thematic analysis, framework analysis, grounded theory) Mixed-methods studies which included a qualitative component of data collection (as described above) and analysis were eligible for inclusion if the qualitative component was clearly identifiable and suitable for extraction	Opinion pieces, commentaries, case studies, guidelines, audits, clinical observational studies, questionnaire studies, RCTs and other quantitative designs
Evaluation	Studies reporting on patient’s attitudes, perspectives and behaviours surrounding QOL in the broadest sense. This encompassed people’s experiences of the condition (symptoms/pain/recovery), experiences of treatment, their physical, mental, emotional, social, daily and professional functioning (effect on working and any financial difficulties), as well as outcome expectations	Studies where a mixed population were included and it was not possible to separately extract the results for our population of interest
Research type	Primary qualitative studies	–

### Study selection and data extraction

Records were downloaded into Endnote (version 9.2) and deduplicated. Due to the volume of records identified by the searches, titles were screened by a single reviewer [divided amongst three people (HL, AB, GO)]. Abstracts and then full texts were independently screened by two researchers against the eligibility criteria and any discrepancies were resolved by discussion with a third researcher.

A data extraction form for the study characteristics of included studies was developed in Microsoft Excel 2002. Information relating to: authors, participant characteristics, study design, method of data collection and method of analysis were extracted by one researcher and checked by a second (HL, AB). Participants and experiences surrounding QOL and author interpretations from the results sections of included studies were imported into NVIVO (version 12) for data extraction and synthesis.

### Quality assessment

Following guidance by the Cochrane Qualitative and Implementation Methods group, the Critical Appraisal Skills Programme [21] checklist was used to assess the quality of included studies. Each article was assessed independently by two researchers (AB, HL) with discrepancies resolved though discussion with a third researcher (AS).

### Data analysis and synthesis

Thematic synthesis was undertaken. Initial coding involved three researchers (AB, HL, GO) independently coding the entirety of the results section (verbatim quotes and author interpretations) of each included study in NVIVO (version 12) [22]. Coding was largely inductive, however familiarisation with the existing literature through screening and discussions with clinical collaborators also influenced code and theme development. A series of roundtable discussions between the three researchers were then held to group common findings from individual studies into broader descriptive themes. Through a process of constant review and refinement these themes were developed into higher order analytical themes, which moved beyond those used by included studies. This was an iterative process achieved through independent and group discussions of the implications of each theme. This process was led by HL with input from AS, AB, CM, CH, HS and GO and resulted in the identification of the two over-arching, conceptual themes that are used to present our findings in the results section: (1) areas of living key to QOL for lower limb reconstruction patients and (2) moving towards a new normal. After synthesis, the results were shared with our patient, public involvement and engagement (PPIE) group which has been convened for the project and included patients at various stages of recovery after a lower limb reconstruction. The PPIE group discussed the findings from the perspectives of their own experiences and ensured that descriptions and phrasings were appropriate for the topic area. The paper has been reported in accordance with the enhancing transparency in reporting the synthesis of qualitative research (ENTREQ) guidelines which can be found in Additional file 3 [23].

## Results

### Search results

**Fig. 2:**
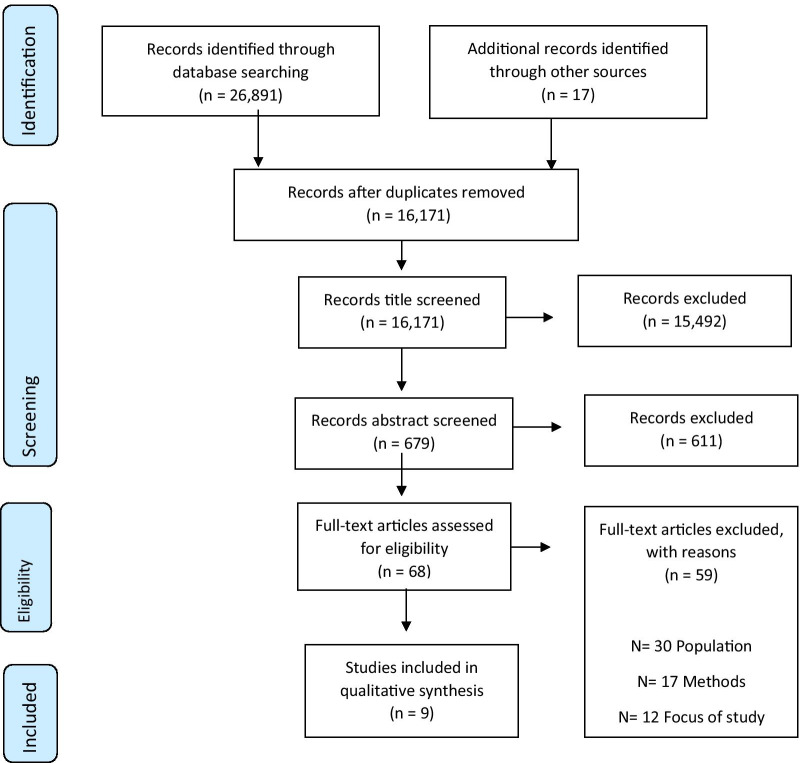
PRISMA flow chart

The search strategies identified 16,171 references (after deduplication). Nine studies representing 124 participants were included in the review (Fig. 2). The characteristics of included studies are provided in Table 2. Five studies were undertaken in the UK [24–28], two in the USA [29, 30], and one each in Australia [31] and Sweden [32]. All studies included patients who had experienced a lower limb fracture. There was considerable variation across studies in the time frame between the patients’ surgery and recruitment into the study (range 5 days to 39 years post-surgery). None of the included studies focussed explicitly on patients QOL. Instead, studies explored patients’ experiences, emotions and daily life post-injury.

**Table 2:** Characteristics of included studies

References	Country	Sample	Method (analytical approach)	Main aim of interviews	Themes identified
Aravind et al. [29]	USA	20 participants with type IIIB or IIIC open tibial fractures. Reconstruction patients received flaps Amputation (n = 9), reconstruction (n = 11) Time since surgery: 2.3–12 years	Semi-structured interviews. (grounded theory)	To explore patient decision making to identify the patients’ preferences and understanding of their injuries	D. Identity E. Emotional wellbeing G. Ability to adapt and adjust
Bernhoff et al. [32]	Sweden	8 participants. 5 reconstruction and 3 amputation after a lower extremity trauma with vascular injury. 4–17 years after surgery	Semi-structured interviews (phenomenology)	To explore how patients experience life, years after severe lower extremity trauma with vascular injury	A. Pain B. Daily lifestyle and functioning C. Income D. Identity E. Emotional wellbeing F. Support G. Ability to adapt and adjust H. Ability to move forwards during recovery
Griffiths and Jordan [24]	UK	9 participants who had a lower limb fracture and undergone surgery. Approximately 3 years after injury	Diaries and semi-structured interviews (Grounded theory)	To investigate patients’ experiences of hospitalisation with lower limb trauma with regards to stressors during recovery, coping methods they put in place and what could change in practice to alleviate stress and augment patients coping strategies	A. Pain B. Daily lifestyle and functioning E. Emotional wellbeing F. Support G. Ability to adapt and adjust H. Ability to move forwards during recovery
McPhail et al. [31]	Australia	12 participants who had previously had an ankle fracture (Distal fibula and/or distal tibia fracture. Seven of these had Open Reduction Internal Fixation) and 6 health professionals who treated ankle fracture patients	In-depth semi-structured interviews. (thematic analysis)	To look into patient experiences of ankle fracture on their everyday activities, work, leisure and how it made them feel	A. Pain B. Daily lifestyle and functioning C. Income D. Identity E. Emotional wellbeing F. Support G. Ability to adapt and adjust H. Ability to move forwards during recovery
Mundy et al. [30]	USA	33 participants who sustained a lower extremity trauma resulting in a limb-threatening lower extremity injury distal to the midfemur which resulted in amputation or required soft-tissue or vascular reconstruction with a local, regional or free tissue transfer for limb salvage. 15 underwent limb salvage or reconstruction, 11 underwent amputation and 7 underwent delayed amputation after failed reconstruction. Less than one to 33 years post injury (mean 6.9 years)	Semi-structured interviews (constant comparison- Interpretive description of transcripts)	To define issues and concepts important to limb salvage patients	B. Daily lifestyle and functioning C. Income D. Identity E. Emotional wellbeing F. Support G. Ability to adapt and adjust H. Ability to move forwards during recovery
Phelps et al. [25]	UK	11 participants with a distal femoral fracture. Patients received intramedullary nails or distal locking plates. Interviewed < 5 months post-surgery. Two patients were interviewed twice	Semi-structured interviews. (thematic analysis)	To understand participants’ experience of the early phase of recovery after a distal femoral fracture	A. Pain B. Daily lifestyle and functioning E. Emotional wellbeing F. Support G. Ability to adapt and adjust
Rees et al. [26]	UK	25 participants who had received reconstructive surgery for an open fracture. Between 24 and 49 months post injury. Gustilo-Anderson II (n = 4) or III (n = 18), IIIc (n = 3)	Individual interviews. (phenomenology)	To understand patients experience of recovery	A. Pain B. Daily lifestyle and functioning C. Income D. Identity E. Emotional wellbeing G. Ability to adapt and adjust H. Ability to move forwards during recovery
Trickett et al. [28]	UK	9 participants were interviewed after an open tibial fracture. Gustilo-Anderson grade I, II, IIIa, IIIb. 1 participant had an amputation and 8 participants received a circular external fixation (n = 5) or an intramedullary nail (n = 3). At least 15 months post-injury (mean injury to interview interval 2.3 years)	Semi-structured interviews. (conventional content analysis)	To explore patients’ personal perspective of their injury, treatment, rehabilitation and psychosocial and financial situations	A. Pain B. Daily lifestyle and functioning C. Income D. Identity E. Emotional wellbeing F. Support G. Ability to adapt and adjust H. Ability to move forwards during recovery
Tutton et al. [27]	UK	20 participants after their first surgical intervention (5–35 days after) due open fracture of the lower limb. Gustilo-Anderson II (n = 4) or III (n-16)	Interviews (phenomenology)	To understand patients’ experiences of injury and recovery	A. Pain B. Daily lifestyle and functioning C. Income D. Identity E. Emotional wellbeing F. Support G. Ability to adapt and adjust H. Ability to move forwards during recovery

### Quality assessment outcome

The quality of included studies was variable with each study having at least one methodological weakness as seen in Additional file 2. Common methodological weaknesses included insufficient detail surrounding ethical issues (7/9 did not meet this criterion) and the relationship between researcher and participants (7/9 did not meet this criterion). In eight studies the information provided allowed us to conclude that the research design was appropriate to address the aims of the research and in seven studies it was evident that the recruitment strategy was appropriate to the aims of the research. In three studies it was unclear if the data analysis was sufficiently rigorous.

## Findings from the thematic synthesis

**Fig. 3:**
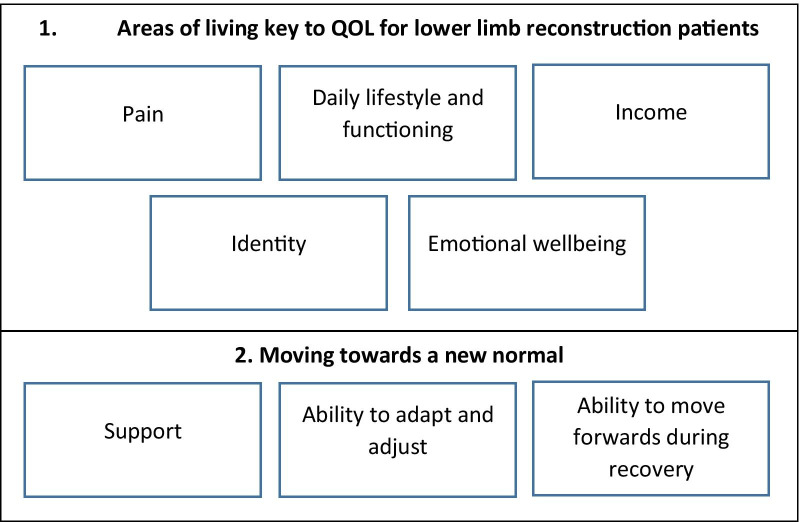
Diagram of the findings from the thematic synthesis

We identified two over-arching themes, which we use to present the findings of our thematic synthesis: (1) areas of living key to QOL for lower limb reconstruction patients and (2) moving towards a new normal (Fig. 3). Although we have chosen to present our findings under these two headings, they should not be viewed in isolation or as separate constructs. Many of the areas of living that are key to QOL which were identified as being affected by limb reconstruction also influence recovery. For example, an individual’s financial situation may affect their ability to continue with rehabilitation and/or physiotherapy if extra costs are associated with this.

### Areas of living key to QOL for lower limb reconstruction patients

#### Pain

For lower limb reconstruction patients, pain was a prominent issue which affected their QOL from the point of injury, immediately post-surgery and throughout their long-term recovery. Those who had experienced traumatic injuries spoke of the pain being almost unbearable. Pain after surgery left patients feeling constantly uncomfortable and medication was seen to just ‘take the edge off’. In relation to recovery and the long term impacts of pain on QOL, pain often hindered patients’ mobility and their ability to engage in daily activities by reducing their strength and energy. Pain also influenced patients’ ability to cope and adapt in the long term. Many grew to live with pain and so made modifications to their daily life to try and avoid or alleviate it. Lastly, some patients suggested that pain could be used as a method for monitoring improvements—some individuals reported how they knew they were getting better when the pain lessened or when pain was replaced by an ache.

Yes there are days that the pain is bad and there have been days where I can’t bear the pain. I’ve been asking for pain killers and I’ve curled up […] to try and deal with the pain. It does have its days of coming and going, the pain [… ] It’s not always just pain, it’s like itching where it’s healing and I can’t itch it which is annoying. There’s aching, itching, pain, throbbing, there’s a burning pain like when you’ve got sunburn, it feels like that on my legs where they took the skin grafts from. Participant 19, Tutton 2018.

#### Daily lifestyle and functioning

Patients reported limitations to their daily activities and lifestyle compared to life before their injury/condition onset. These limitations related to normal day-to-day activities that are usually taken for granted such as getting dressed, moving around the home, doing housework, cooking or going to the toilet. Patients also reported difficulties sleeping due to pain or discomfort which impacted daily living if they were tired and not well rested. Patients often described being unable to participate in their usual hobbies, or pastimes due to poorer physical function and movement. For example, a number of patients struggled to play with their grandchildren or help with their care like they used to. Some missed the sport they used to be able to play, whereas others missed social activities such as going to the pub socialising with friends and engaging in community events.

I can’t play with my grandchildren the way I would like to, I can’t run around and play in the woods and fields. I can’t sit down in the sandbox. So certainly I’d like to be more active you feel like a really old grandma, even though you are not. Participant 4, Bernhoff 2016.

Lengthy recovery times and long periods with poor mobility meant that the impact of reconstructive surgery on functional and physical impairment was a long term issue, which had knock-on effects on patients’ physical and mental health. Even after they had been told they were ‘recovered’ many patients felt their limb to be unstable and weak, that they were unable to achieve a full range of movement and consequently were cautious using it. As a result, deteriorating physical health also had further knock on effects on patients’ ability to recover and regain their mobility.

But it’s the standing, the standing and putting the weight on that side and I am just no confidence in myself at all. Participant 9, Phelps 2019.

#### Income

Recovery or ongoing functional impairment negatively impacted patients’ careers and their ability to return to work in the same capacity, if at all. This exacerbated feelings of hopelessness and worthlessness and created a further financial impact for patients. Some struggled with not being able to work whilst they recovered, even if they would eventually be able to return to work as normal. Some took unpaid leave, had to leave their job, or were made redundant. Those with zero hour contracts were concerned about their future hiring potential. Some patients were able to go back to their old job with modifications in their hours or tasks whereas others looked for new jobs and found satisfaction in trying something new.

You want to live for the future now because that’s what you’ve got in front of you but you worry about what it’s going to bring, not only physically but financially as well. You have no idea what your pay-out is going to be and so my life is in somebody else’s hands. There is that horrible thought that you may have to go back out to work, force yourself to work because financially you can’t exist for the rest of your life. Participant 13, Rees 2019.

For those who could not work during their treatment and recovery, loss of income negatively impacted on their QOL and their ability to recover—irrespective of whether they lived in a country with a public or private healthcare service. Patients often relied on their savings to support their recovery, with some unable to continue taking their medication because they did not have the money to pay for it once the hospital prescribed medication ran out. Finances also impacted recovery if physiotherapy was an extra cost or if patients were required to purchase specific equipment.

An associated impact was the use of savings to compensate for reduced income or greater expenses (including expenditure on healthcare costs). …Patients reported financial impacts of mixed severity. Participants frequently reported reduced income as the primary financial impact. Many participants stated they were “out of work so it affected money” (p11) or suffered “loss of income as (I was) unable to work at full capacity for some months” (p2). Author quote, McPhail 2012.

Participants experienced distress due to the impact their lack of finances could or was having on their family. This included not having enough money for food, new clothes, or to send their children to school. Some depleted their savings as they could not work, and others had to sell belongings to pay for food. Those that could be financially supported by others felt guilty and pathetic that they had to do so.

#### Identity

The functional and cosmetic impairments associated with lower limb reconstruction also had negative impacts on a patient’s identity. These included not being able to wear the clothes they used to wear and having to stop doing jobs they loved or pastimes that were integral to their sense of self. More specifically, some patients reported dissatisfaction, shame or embarrassment at the way their leg looked after surgery and were worried what others would think when they saw it. A number of patients had gained weight during their recovery which further negatively impacted their body image, clothing choices and their functionality. Some modified what they wore so that they could hide the leg, choosing not to wear shorts. However, many countered this by saying that overall they were just happy they still had their leg and some exposed the leg despite the questions or looks they got. Overall, many had ‘come to terms’ with the way their leg now looked.

I don’t look at it as my leg anymore… It is like the legs belongs to somebody else they don’t particularly belong to me. Participant 19, Rees 2019.

A loss of identity made patients feel vulnerable, and it was very important to patients to be seen as a person while they were in hospital and during recovery, rather than just a leg. A few patients spoke very strongly about their leg being a part of them that they could not bear the thought of losing. These patients believed that without their leg they would have had nothing to work towards, would have struggled emotionally and had suicidal thoughts.

“[Amputation] would have crushed me, because now I have nothing to work for. It can’t possibly be put back on now. The fact that they did reconstruct it gave me a lot of willpower to want to do what I could.” -Male, 53 years, reconstruction, 10 years post-injury. Participant quote, Aravind 2010.

#### Emotional well-being

Patients felt anxious before surgery as well as during recovery. Anxiety often related to future worries, as patients were concerned about how they would cope, who would support them, how they would support others, how long their recovery process would take and what it would entail. Patients experienced a number of fears that related to surgery and recovery which included a fear of: the surgery not working and/or an incomplete recovery; not waking up, being paralysed after surgery; re-injury; not being able to walk; exercise or work as well as ongoing fears of losing their leg. Patients also experienced uncertainty regarding their future and what this would look like.

“All patients described some aspect of fear during their recovery, regardless of initial injury circumstance, clinical course or adequacy of clinically perceived recovery. Fear was a prominent term used in all interviews and appeared to persist through to the final stages of recovery, even when patients had completed their treatment and rehabilitation” Author quote, Trickett 2012.

A number of patients reported experiencing low mood and depression after their surgery and during recovery. This was usually associated with the slow process of recovery and feeling isolated or lonely. Patients experienced particular frustration at how long recovery took and how long it took for them to regain their functionality and mobility. Many felt bored with not being able to move around and/or not being able to help around the house. Additionally, many patients felt vulnerable before and during surgery, realising that their life was in someone else’s hands. Despite their experiences patients were very grateful that they did not lose their leg and that they were alive and in a position where they would be able to recover.

It wasn’t until I got right down to the anaesthetics room that the penny dropped and then I was like a big girl’s blouse [idiom: not brave or confident]because I didn’t have the wife there or anybody there just two strangers and I felt lonely and vulnerable and basically my life is in their hands. Participant 3, Tutton 2018.

### Moving towards a new normal

#### Support

##### Support from healthcare professionals

The support patients received whilst in hospital was a crucial ‘first step’ in their recovery. Strong supportive networks were very important to patients and appeared to lessen the negative impact recovery had on patients’ QOL. However, patients had mixed experiences regarding how supported they felt whilst in hospital. Patients felt supported when a staff member made them feel more comfortable, kept them informed, made them feel normal (i.e. more than just a patient with a lower limb condition), and reassured them that their situation was improving. Being treated as a ‘normal’ person was important for patients, who often felt as though staff talked about their leg and their diagnosis rather than to them as individuals. Having confidence in the treating clinician comforted patients that they were in the best hands and made them feel safe. Clinical staff also provided an authority figure and ensured that patients trusted the information that was given to them about their treatment and recovery. For instance, those that were awake during surgery appreciated being kept informed about what was being done to them. Additionally, hearing from staff about their recovery also gave patients confidence during rehabilitation and ensured they had realistic expectations about their recovery. Conversely, not feeling informed was distressing to patients and resulted in a number of knock on consequences including: worry, vulnerability, not feeling valued/cared for and influenced patients’ compliance with medication or physiotherapy. Whilst some patients felt uninformed about their treatment and recovery due to issues with their capacity during clinical consultations, others reported being unsupported by hospital staff. For example, one patient heard staff laughing and joking in the operating room before being anaesthetised and was concerned about their professionalism and the ability of the clinical team to care for them.

I recognised [name of nurse] before I went to theatre. . . and her being there, and knowing her helped me a lot actually. Even though we had not talked, it is as if we knew each other, there is a bit of a bond there, it helped me be strong to a certain extent. Participant 9, Griffiths 1998.

Patients shared different opinions on discharge, some felt they had been discharged from hospital too soon, whilst others felt it was the right time. Opinion was also divided on the level of in-patient rehabilitation that was provided and whether this was of benefit—some gained confidence from this support, whilst others either did not find it useful or were not offered it. Patients reported receiving support as an outpatient from a range of sources including: physiotherapists, local GPs and/or hospital staff for follow-up appointments. However, the amount and type of outpatient support received was variable. Some patients felt that there was a lack of support for emotional problems, whilst others reported having a great, supportive physiotherapist who greatly aided their recovery. Additionally, although some patients reported receiving medication support from their local GP, others experienced issues with seeking support after being discharged—often being unsure of how to access support as an outpatient. Similarly, whether participants were provided with physiotherapy equipment to use at home or even offered physiotherapy varied. Patients often felt that their poor function was due to a lack of instructions or training with crutches. In all studies, a lack of outpatient support was seen as detrimental to recovery and QOL.

Once they were discharged from hospital, however, participants described receiving delayed or no support from physiotherapists. Lack of support with rehabilitation could impair some patients’ confidence who were reluctant to move on their own. Author quote, Phelps 2019.

##### Supportive relationships and strains

Many patients had strong, supportive relationships with friends and family which were highly valued and influential in aiding adaptation after a lower limb reconstruction. These relationships took the form of partners, parents, children or support networks such as churches, cleaners and other paid house workers. In a few circumstances these relationships had come under strain which in some cases was due to the caring load negatively impacting on a partner’s ability to work. One consequence of a lack of supportive networks was feeling lonely and socially isolated; this was a particular issue for patients whose lack of mobility meant they were housebound. However, even patients who had support networks in place reported feeling like a burden on their families and felt guilty for needing to rely on others for support. This was worse for those who were normally the caregiver of the family. This feeling of being a burden further negatively impacted on patients’ well-being and QOL during recovery.

You feel a bit like a passenger in it all because you’re on the outside looking in and you think you’re being a bit of a burden on everyone. At the time I found it quite hard to almost tell people that’s how I felt, I feel this, I feel a bit worthless. Participant 18 quote, Rees 2019.

#### Ability to adapt and adjust

##### Making modifications

Patients discussed making modifications to their lifestyle as a way of adapting and adjusting to life after limb reconstruction surgery. For some patients these modifications were negative and resulted in poorer physical or mental health. For others, modifications had a positive effect and aided their recovery, well-being and their ability to move towards a new normal.

However, with help from others or by making adaptations, some participants were able to manage and overcome the restrictions imposed by their injury. For example, they ironed sitting down, washed dishes from a stool or hoovered from their wheel chair, as standing for long without support was a struggle. Their ability to adapt to their impaired mobility suggests a degree of resilience. Author quote, McPhail 2012.

Being able to adapt and adjust to their situation in a way that enabled them to still participate in the things they enjoyed doing or saw as part of their role were key to a higher QOL after surgery. These related to things such as sitting to do the ironing or wash dishes, reducing working hours or location.

I learned to adapt it’s like I had a pair of leggings made that were sort of Velcro on the side and things like that, wearing clothes wasn’t a problem. Participant 5 quote, Trickett 2012.

I had a wheelchair and I used to Hoover because I didn’t want it to get in the way of my life. Even to light the cooker I’d get down on a bean bag, light the cooker and then push myself back up on to my wheelchair. Participant 7 quote, Trickett 2012.

Many patients reported a reduced ability to exercise. Those who adapted to this or who were content with their ‘new normal’ experienced positive side effects from these modifications such as joy in finding a new way to exercise, finding a physical activity that actually helped their recovery, losing weight by being active or finding a new hobby that was not sport related. For some, being able to do a modified version of their previous activities was enough to bring them joy, but for others it was not. For instance, a number of participants voiced fears of re-injury, which had knock on consequences on their daily activities often resulting in them not doing activities or doing them differently to account for the risk they perceived. Patients reported that they were more cautious about how they moved and what they did, with a fear of falling or re-injury often at the back of their mind. For some a fear of injury was seen to negatively impact QOL since it hindered a return to normality.

##### Control

Some patients were assertive, and demonstrated that they had control over their situation, life and recovery. Positively, control was associated with a return to normality and with coping as a way of recovering. Patients who felt in control were more likely to report favourable outcomes.

“After their initial shock, our respondents began to regain a sense of control; thus, they began their `return to normal'. Appraisal and coping at the time of injury were in the form of problem focused coping, in order to reduce pain. For example, subjects 4 and 8 tried to prevent any movement to pre-empt the onset of acute pain” Author quote, Griffiths 1998.

Many patients put strategies or mechanisms in place to help them cope with their situation which were in turn perceived to positively impact their recovery and QOL. These strategies included laughing and making a joke of their situation, making problem-focused modifications such as moving as little as possible to avoid the pain, controlling their medication, focusing on the positives of physiotherapy, focusing on their faith, and having realistic expectations for their recovery e.g. it’s ok to be a bit stiff. Patients felt they needed to stay strong and show resilience, although in some cases this was considered emotionally draining in the long term. Others used negative mechanisms such as alcohol and dysfunctional eating habits to cope with their pain and recovery, which often resulted in worse emotional well-being and poorer body image.

##### Motivation towards recovery

Whether patients were motivated towards recovery also influenced their ability to adapt and adjust after treatment. Patients spoke of the positive impact of willpower and determination for their recovery. Motivation was also linked to adherence with physiotherapy and with pushing through pain barriers or challenging situations. Many were motivated to adhere to their rehabilitation plan to avoid any negative impacts on their future functionality. Being motivated was also important in that it encouraged patients to set goals, prioritise recovery and make sure they had enough time to do their physiotherapy exercises. Some patients were motivated by the progress of others and used them as a benchmark to work towards. Similarly, patients enjoyed having something to work towards such as regaining their independence, maintaining or regaining fitness, going home, being able to run again or walk a certain distance. Those who felt optimistic about their recovery and felt empowered to get better were more likely be motivated to recover. Factors that decreased motivation included a lack of equipment, the length of the rehabilitation process, the repetition of exercise and a lack of the perceived effectiveness of the exercises. Those who were less motivated were also less adherent to their physiotherapy programs.

My wife would phone me and she’d say where are you? I said I’m on the bus to town. What? You’ve got a broken leg. Yes, yes I’ll be fine don’t worry about it. . .. But it was my drive to be independent again like. Participant 2 quote, Trickett 2012.

#### Ability to move forwards during recovery

As recovery continued, patients’ responses indicated a return to normality or the development of a new normal. This often occurred when participants had managed to integrate their disability into normal life. Patients experienced the return to normal as a long process, often including a re-orientation of the meaning of life itself, finding a new normal for themselves and focusing more on what is important to them. Some felt that they had learnt a lot about themselves through their treatment and recovery. Many found that a lot of their negative experiences and feelings had resolved as they were able to return to previous activities. What was classed as a return to normality differed for each individual but mostly included being able to undertake/perform tasks or activities they had enjoyed doing in the past.

“Participants discussed accepting their injuries and moving forward (e.g., as good as it’s going to get, it is what it is, move forward, new normal, part of life now);…and a positive impact from the injury (e.g., appreciate small things in life, better person, changed priorities, gratitude, patience, stronger, think of others).” Author quote, Mundy 2020.

Many patients had accepted their situation and had come to terms with their treatment and the long term recovery process. However, for some patients this acceptance seemed begrudged. They were not fully content but felt that constant worrying and focusing on the negative aspects of their situation would just make things unnecessarily worse for themselves. Having concerns for the future and how life would be changed following recovery was a common element of moving forwards. In particular, people worried about how their injury would impact their ability return to a normal routine, engage in their usual pastimes and hobbies, their ability to return to work, care for their family and the long term impact on their finances. Some were very concerned that in the long term they might need an amputation if recovery did not go well.

Yeah, I’ve thought a lot about it of course, how they managed to save it, and I’m happy I still have my leg, though at first I thought having these scars was just crap. But I’ve come to terms with it; I thought about it a lot. At first it was really difficult, with these scars in particular, but you always have to remember there are people who are worse off. Participant 5 quote, Bernhoff 2016.

## Discussion

This review synthesised qualitative evidence from nine studies which represents the experiences of 124 individuals from four countries to explore what is important to patients with regards to QOL after a lower limb reconstruction. The included studies were of variable quality and included patients who were at different stages of recovery and had received lower limb reconstruction due to a variety of traumatic injuries.

We identified a number of areas of living that are key to QOL for patients after a lower limb reconstruction: pain, daily lifestyle and functioning, income, identity, emotional well-being, support, ability to adapt and adjust and ability to move forwards. The review also highlighted the important role that an individual’s recovery and the factors that promote or inhibit their journey towards a ‘new normal’ plays on QOL. A key finding of this review is that the way in which lower limb reconstruction affects an individual’s QOL and their recovery is complex and is influenced by a range of inter-related factors, which will affect patients to varying degrees depending on their individual circumstances. For example, the extent to which treatment and recovery from a lower limb condition negatively affected patients’ QOL (compared to baseline) was likely to be influenced by the support they received from others, the pain they experienced, their overall emotional well-being and their ability to adapt and cope with various lifestyle changes following reconstruction. Additionally, an individual’s ability to adapt and adjust to a ‘new normal’ is in itself likely to be mediated by the strength of the relationships underpinning their support network and the impact of reconstruction on areas of living such as family income. Supporting this point, our PPIE group believed that strong support networks were key to maintaining QOL during recovery from surgery. To further add to this complexity, our review found that what patients consider to be important with regards to their QOL may change over the course of their treatment and recovery. For instance, pain and physical functioning were more important to patients before and immediately following reconstruction surgery, whilst their ability to adapt and make lifestyle modifications played a more prominent role later in their journey towards a new ‘normal’. Resultantly, it is important that a PROM is sensitive to changes across the patient recovery period.

Our findings are consistent with the broader literature in this area which has identified a range of social, medical, behavioural and physiological factors that patients experience following lower limb reconstruction [33–35]. Findings from research with patients who experienced a lower limb injury which did not require reconstruction showed they experienced similar impacts to their QOL; the injury impacted their daily functioning and lifestyle, pain, clinical and personal support networks and rehabilitation played important roles in their recovery [36–39]. Similarities with our findings can also be seen in the psychological and social factors experienced during recovery in patients who experienced a traumatic knee related injury [40], in particular a fear of re-injury, a changed identity and emotional well-being [41, 42]. One difference between the experiences of different groups of patients however seems to be the negative impact of pain and the importance of the role of support. These appear to play a more vital role for patients after a lower limb reconstruction compared to those undergoing knee surgery. In contrast, those who injure themselves playing sport appear to be more strongly driven to regain pre-injury levels of physical activity after recovery as well as being more likely to make modifications to enable them to achieve this [43–45]. However, it is possible that these traits may also be seen in lower limb reconstruction patients who experienced high levels of activity pre-injury.

Previous research specific to this area has paid relatively little attention to exploring the inter-related nature of the various domains of QOL which are affected by lower limb reconstruction, the influence of recovery and support on QOL and how these influencing factors may change over time. Future research is therefore required to evaluate whether existing PROMs take into account the various areas of living which we have identified here as being key to QOL that are affected by lower limb reconstruction. More specifically, it is important that future qualitative research and future PROMs consider the relationship between QOL and recovery and are sensitive to potential temporal influences across the patient’s recovery period if they are to inform clinical and patient decision-making. Future qualitative research may also wish to explore differences between types of lower limb reconstruction patients e.g. acute trauma, elective patients, those with congenital disorders or deformities or those experiencing malunion or non-union as it is possible that this could influence QOL. We have been intentionally broad in what we have sought to capture in this preliminary qualitative evidence synthesis and have not put constraints on the data we have extracted and coded from included studies in order to avoid imposing our assumptions on what is important to patients. Therefore, we have captured items related to patient experience of care and potential mediators of QOL which may not be relevant for a PROM being utilised to assess the effectiveness of surgical interventions on function QOL. The specific function and proposed uses for any new tool requires careful consideration as this will determine the items that need to be captured in the tool.

## Strengths and limitations

The review was conducted in line with recently published guidance for conducting QES by the Cochrane Qualitative and Implementation Methods group [17]. To ensure a robust and systematic approach our synthesis was undertaken by three health services researchers (HL, AB, GO), without previous experience of PROM development or lower limb reconstruction. This was complemented by discussion with the PPIE group and roundtable discussion with researchers with experience of conducting research in orthopaedic surgery and a consultant orthopaedic surgeon.

Whilst our searches were comprehensive and were undertaken by an experienced information specialist, it is possible that we failed to find some relevant studies. Additionally, we only included studies published in English and so the generalisability of our findings may be limited. Our search terms were kept as broad as possible and included qualitative study design filter terms to minimise the risk of missing studies. A consequence of our broad search strategy was that a large number of studies were excluded at the title stage. We put the emphasis on sensitive searches to minimise the risk of missing relevant studies as much as possible given the broad population of lower limb reconstruction, the limitations with the indexing of qualitative studies and the wide variety of terms that are used to describe qualitative methods. Studies were excluded at this stage predominantly due to their population not being eligible (children, amputee, knee or hip replacement) or methods used (clinical observations, clinical case studies, quantitative studies). There is a current debate within the QES field regarding the optimal method to search and retrieve qualitative evidence for systematic reviews. Some believe that systematic searching is not appropriate for qualitative synthesis due to inadequate indexing and unclear titles [46]. Others opt for search strategies with an inclusive focus on broad qualitative terminology. This was our chosen approach through the use of a qualitative search strategy that included thesaurus terms and both specific and broad free-text terms based on an evaluation of qualitative search strategies by Shaw et al. [18]. As such, our search was systematic and broad with greater focus on sensitivity than specificity. However, we preferred this approach to the alternative of potentially missing relevant articles. Although our search criteria included patients with congenital disorders that required lower limb reconstruction we did not identify any eligible studies with these patients. This is likely to be because reconstruction for these patients is often undertaken in childhood and the synthesis did not include children under the age of 16.

## Conclusion

The findings of this QES suggest that the way in which lower limb reconstruction affects a patient’s QOL is complex, may change over time and is linked to their recovery. We will use these findings to develop a conceptual framework which will identify and map what is important to patients during or after undergoing reconstructive surgery for a lower limb condition and identify elements important to include in a PROM. Qualitative work is currently being undertaken with a variety of lower limb reconstruction patients (type of condition/injury and treatment) and health professionals (surgeons, physiotherapists and nurses) from across the UK to aid the development of the conceptual framework; our findings will be shared in due course. Future research is required to establish whether the range of factors identified in this review that affect QOL following lower limb reconstruction are captured by existing PROMs. Depending on the outcome of this work, a new PROM for patients following lower limb reconstruction may be required.

## Supplementary Information

**Additional file 1**. Appendix 1: Search strategies.

**Additional file 2**. Appendix 2: Quality assessment outcome table.

**Additional file 3**. Appendix 3: ENTREQ checklist.

## Data Availability

All data generated or analysed during this study are included in this published article [and its supplementary information files].

## Abbreviations

QES: Qualitative evidence synthesis
QOL: Quality of life
PROM: Patient reported outcome measures
ENTREQ: Enhancing transparency in reporting the synthesis of qualitative research
CASP: Critical appraisal skills programme
PPIE: Patient, public, involvement and engagement
