# Supplementary material for: Exploring what is important to patients with regards to quality of life after experiencing a lower limb reconstructive procedure: a qualitative evidence synthesis
Source: Health Qual Life Outcomes. 2021 May 31;19:158. doi: 10.1186/s12955-021-01795-9 (PMC8166062; doi:10.1186/s12955-021-01795-9)
Supplement: Supplementary file 1 — Additional file 1. Appendix 1: Search strategies. [file 12955_2021_1795_MOESM1_ESM.docx]

Appendix 1. Search strategies.

**MEDLINE ALL**

via Ovid <http://ovidsp.ovid.com/>

1946 to November 13, 2020

Search date: 13^th^ November 2020

Records retrieved: 7589

| 1 | Ankle Fractures/ | 1571 |
| --- | --- | --- |
| 2 | femoral fractures/ | 16559 |
| 3 | tibial fractures/ | 15344 |
| 4 | 1 or 2 or 3 | 30939 |
| 5 | ((fractur$ or trauma$) adj3 (lower extremit$ or lower limb$)).ti,ab. | 3344 |
| 6 | ((fractur$ or trauma$) adj3 (leg or legs or thigh$ or knee or knees or shin or shins or shinbone$ or foot or midfoot or hindfoot or feet or ankle or ankles or anklebone$ or pilon or heel or heels or heelbone$ or toe or toes)).ti,ab. | 10235 |
| 7 | ((fractur$ or trauma$) adj3 (femur$ or femoral or tibia$ or fibula$ or patella$ or kneecap$ or knee cap$)).ti,ab. | 35926 |
| 8 | ((fractur$ or trauma$) adj3 (metatarsal$ or metatarsus or metatarsi or tarsus or tarsi or tarsal or tarsals or cuneiform or cuboid or navicular or calcane$ or talus or astragalus or tali or bimalleol$ or malleol$ or trimalleol$)).ti,ab. | 5670 |
| 9 | ((Pott$ or Cotton$) adj fractur$).ti,ab. | 30 |
| 10 | or/5-9 | 51118 |
| 11 | lower extremity/ or exp foot/ or knee/ or leg/ or thigh/ | 147052 |
| 12 | leg injuries/ or ankle injuries/ or foot injuries/ or knee injuries/ | 39714 |
| 13 | exp Leg Bones/ | 50937 |
| 14 | exp Foot Bones/ | 17859 |
| 15 | 11 or 12 or 13 or 14 | 236559 |
| 16 | (lower extremit$ or lower limb$).ti,ab. | 103833 |
| 17 | (leg or legs or thigh$ or knee or knees or shin or shins or shinbone$ or foot or midfoot or hindfoot or feet or ankle or ankles or anklebone$ or pilon or heel or heels or heelbone$ or toe or toes).ti,ab. | 430698 |
| 18 | (femur$ or femoral or tibia$ or fibula$ or patella$ or kneecap$ or knee cap$).ti,ab. | 254437 |
| 19 | (metatarsal$ or metatarsus or metatarsi or tarsus or tarsi or tarsal or tarsals or cuneiform or cuboid or navicular or calcane$ or talus or astragalus or tali or bimalleol$ or malleol$ or trimalleol$).ti,ab. | 39443 |
| 20 | 15 or 16 or 17 or 18 or 19 | 758756 |
| 21 | Fractures, Bone/ | 64824 |
| 22 | 20 and 21 | 18839 |
| 23 | (fractur$ or trauma$).ti,ab. | 592841 |
| 24 | 15 and 23 | 31587 |
| 25 | exp Fracture Dislocation/ | 2260 |
| 26 | Fractures, Avulsion/ | 172 |
| 27 | Fractures, Closed/ | 3325 |
| 28 | Fractures, Compression/ | 2359 |
| 29 | Fractures, Malunited/ | 1630 |
| 30 | Fractures, Multiple/ | 171 |
| 31 | Fractures, Open/ | 5458 |
| 32 | Fractures, Stress/ | 3392 |
| 33 | exp Fractures, Ununited/ | 10802 |
| 34 | Intra-Articular Fractures/ | 1275 |
| 35 | Periprosthetic Fractures/ | 1149 |
| 36 | Fracture Healing/ | 13586 |
| 37 | Crush Injuries/ | 187 |
| 38 | (crush$ adj2 (injur$ or trauma$ or fractur$)).ti,ab. | 3916 |
| 39 | Fractures, Comminuted/ | 2547 |
| 40 | or/25-39 | 45719 |
| 41 | 20 and 40 | 19106 |
| 42 | exp Multiple Trauma/ | 12958 |
| 43 | (polytrauma$ or poly-trauma$ or multitrauma$ or multi-trauma$).ti,ab. | 5055 |
| 44 | ((complex$ or complicat$ or severe$ or severity or serious or major or multiple) adj2 (fractur$ or trauma$)).ti,ab. | 39942 |
| 45 | or/42-44 | 51884 |
| 46 | 20 and 45 | 7928 |
| 47 | 4 or 10 or 22 or 24 or 41 or 46 | 88807 |
| 48 | ((bone$ or fractur$) adj3 (heal or heals or healed or healing)).ti,ab. | 23467 |
| 49 | ((bone$ or fractur$) adj3 (union$ or nonunion$ or non union$ or ununite$)).ti,ab. | 9006 |
| 50 | ((bone$ or fractur$) adj3 (malunion$ or mal union$ or deform$)).ti,ab. | 4843 |
| 51 | (osteomyelitis or ((bone$ or fractur$) adj3 infect$)).ti,ab. | 30751 |
| 52 | exp Osteomyelitis/ | 22961 |
| 53 | or/48-52 | 71686 |
| 54 | 20 and 53 | 22419 |
| 55 | ((femur$ or femoral or tibia$ or fibula$ or patella$ or kneecap$ or knee cap$) adj3 (heal or heals or healed or healing)).ti,ab. | 1493 |
| 56 | ((metatarsal$ or metatarsus or metatarsi or tarsus or tarsi or tarsal or tarsals or cuneiform or cuboid or navicular or calcane$ or talus or astragalus or tali or bimalleol$ or malleol$ or trimalleol$) adj3 (heal or heals or healed or healing)).ti,ab. | 120 |
| 57 | ((femur$ or femoral or tibia$ or fibula$ or patella$ or kneecap$ or knee cap$) adj3 (union$ or nonunion$ or non union$ or ununite$)).ti,ab. | 1878 |
| 58 | ((metatarsal$ or metatarsus or metatarsi or tarsus or tarsi or tarsal or tarsals or cuneiform or cuboid or navicular or calcane$ or talus or astragalus or tali or bimalleol$ or malleol$ or trimalleol$) adj3 (union$ or nonunion$ or non union$ or ununite$)).ti,ab. | 162 |
| 59 | ((femur$ or femoral or tibia$ or fibula$ or patella$ or kneecap$ or knee cap$) adj3 (malunion$ or mal union$ or deform$)).ti,ab. | 1975 |
| 60 | ((metatarsal$ or metatarsus or metatarsi or tarsus or tarsi or tarsal or tarsals or cuneiform or cuboid or navicular or calcane$ or talus or astragalus or tali or bimalleol$ or malleol$ or trimalleol$) adj3 (malunion$ or mal union$ or deform$)).ti,ab. | 567 |
| 61 | ((femur$ or femoral or tibia$ or fibula$ or patella$ or kneecap$ or knee cap$) adj3 (osteomyelitis or infect$)).ti,ab. | 1781 |
| 62 | ((metatarsal$ or metatarsus or metatarsi or tarsus or tarsi or tarsal or tarsals or cuneiform or cuboid or navicular or calcane$ or talus or astragalus or tali or bimalleol$ or malleol$ or trimalleol$) adj3 (osteomyelitis or infect$)).ti,ab. | 393 |
| 63 | or/55-62 | 7914 |
| 64 | Limb Salvage/ | 4269 |
| 65 | ((limb or limbs) adj3 (restor$ or reconstruct$ or salvag$)).ti,ab. | 9060 |
| 66 | Ilizarov Technique/ | 1320 |
| 67 | External Fixators/ | 5893 |
| 68 | Orthopedic Fixation Devices/ | 5035 |
| 69 | Fracture Fixation/ | 18605 |
| 70 | Bone Lengthening/ | 2237 |
| 71 | Ilizarov.ti,ab. | 2235 |
| 72 | (external adj2 (fixat$ or frame$ or cage$)).ti,ab. | 10257 |
| 73 | (circular adj2 (fixat$ or frame$ or cage$)).ti,ab. | 670 |
| 74 | Taylor Spatial Frame$.ti,ab. | 213 |
| 75 | TSF.ti,ab. | 1290 |
| 76 | True Lok Hex.ti,ab. | 0 |
| 77 | TLHex.ti,ab. | 0 |
| 78 | or/64-77 | 46215 |
| 79 | 20 and 78 | 21726 |
| 80 | 47 or 54 or 63 or 79 | 111639 |
| 81 | exp qualitative research/ | 58758 |
| 82 | "Surveys and Questionnaires"/ | 476366 |
| 83 | Self Report/ | 34138 |
| 84 | exp Attitude/ | 582851 |
| 85 | Focus Groups/ | 30865 |
| 86 | Ethnology/ | 1589 |
| 87 | discourse analysis.mp. | 1937 |
| 88 | content analysis.mp. | 29919 |
| 89 | ethnographic research.mp. | 1002 |
| 90 | ethnological research.mp. | 7 |
| 91 | constant comparative method.mp. | 1616 |
| 92 | qualitative validity.mp. | 23 |
| 93 | purposive sample.mp. | 3600 |
| 94 | observational method$.mp. | 793 |
| 95 | field stud$.mp. | 15743 |
| 96 | theoretical sampl$.mp. | 720 |
| 97 | phenomenology.mp. | 9556 |
| 98 | phenomenological research.mp. | 488 |
| 99 | life experience$.mp. | 5833 |
| 100 | or/81-99 | 1046699 |
| 101 | interview$.mp. or interviews/ or Interviews as Topic/ | 401496 |
| 102 | qualitative.mp. | 258167 |
| 103 | or/101-102 | 566627 |
| 104 | 100 or 103 | 1414677 |
| 105 | ethnograph$.mp. | 11350 |
| 106 | phenomenol$.mp. | 27428 |
| 107 | grounded theory.mp. | 12206 |
| 108 | (grounded adj (theor$ or study or studies or research or analys?s)).mp. | 12390 |
| 109 | (emic or etic or hermeneutic$ or heuristic$ or semiotic$).mp. | 17871 |
| 110 | (data adj1 saturat$).tw. | 1328 |
| 111 | participant observ$.tw. | 4664 |
| 112 | (action research or cooperative inquir$ or co operative inquir$ or co-operative inquir$).mp. | 4453 |
| 113 | (field adj (study or studies or research or observation$)).tw. | 20606 |
| 114 | theoretical sampl$.mp. | 720 |
| 115 | (purpos$ adj4 sampl$).mp. | 15915 |
| 116 | (focus adj group$).mp. | 54275 |
| 117 | (account or accounts or unstructured or open-ended or open ended or text$ or narrative$).mp. | 666800 |
| 118 | (life world or life-world or conversation analys?s or personal experience$ or theoretical saturation).mp. | 15594 |
| 119 | lived experience$.tw. | 6359 |
| 120 | (theme$ or thematic).mp. | 116350 |
| 121 | (observational adj (method$ or research or stud$)).mp. | 177563 |
| 122 | questionnaire$.mp. | 770093 |
| 123 | content analysis.mp. | 29919 |
| 124 | thematic analysis.mp. | 21295 |
| 125 | discourse analys?s.mp. | 1975 |
| 126 | ((discourse$ or discurs$) adj3 analys?s).tw. | 2331 |
| 127 | (constant adj (comparative or comparison)).mp. | 4822 |
| 128 | narrative analys?s.mp. | 1347 |
| 129 | or/105-128 | 1769110 |
| 130 | 104 or 129 | 2443101 |
| 131 | 80 and 130 | 6327 |
| 132 | survey$.ti,ab. | 664521 |
| 133 | (mixed method$ or multimethod$ or multi-method$ or multi method$).mp. | 27917 |
| 134 | (patient$ adj5 (attitude$ or belief$ or believ$ or experienc$ or opinion$ or perceiv$ or perception$ or perspective$ or preference$ or view or views or viewpoint$)).ti,ab. | 281129 |
| 135 | or/132-134 | 936296 |
| 136 | 80 and 135 | 3342 |
| 137 | 131 or 136 | 8852 |
| 138 | exp animals/ not humans/ | 4762182 |
| 139 | (rat or rats or mouse or mice or rodent or rodents or swine or porcine or murine or sheep or lamb or lambs or ewe or ewes or pig or pigs or piglet or piglets or sow or sows or rabbit or rabbits or kitten or kittens or dog or dogs or puppy or puppies or monkey or monkeys or horse or horses or foal or foals or equine or calf or calves or cattle or heifer or heifers or hamster or hamsters or chicken or chickens or livestock or goat or goats).ti. | 2154092 |
| 140 | 138 or 139 | 5135197 |
| 141 | 137 not 140 | 8519 |
| 142 | limit 141 to english language | 7589 |

**EMBASE**

via Ovid <http://ovidsp.ovid.com/>

1974 to 2020 November 12th

Search date: 13^th^ November 2020

Records retrieved: 12876

| 1 | exp ankle fracture/ | 6763 |
| --- | --- | --- |
| 2 | exp femur fracture/ | 29584 |
| 3 | exp tibia fracture/ | 16659 |
| 4 | exp foot fracture/ | 3697 |
| 5 | knee fracture/ | 724 |
| 6 | patella fracture/ | 1716 |
| 7 | leg fracture/ | 2434 |
| 8 | exp fibula fracture/ | 1755 |
| 9 | or/1-8 | 56024 |
| 10 | ((fractur$ or trauma$) adj3 (lower extremit$ or lower limb$)).ti,ab. | 4126 |
| 11 | ((fractur$ or trauma$) adj3 (leg or legs or thigh$ or knee or knees or shin or shins or shinbone$ or foot or midfoot or hindfoot or feet or ankle or ankles or anklebone$ or pilon or heel or heels or heelbone$ or toe or toes)).ti,ab. | 12157 |
| 12 | ((fractur$ or trauma$) adj3 (femur$ or femoral or tibia$ or fibula$ or patella$ or kneecap$ or knee cap$)).ti,ab. | 41519 |
| 13 | ((fractur$ or trauma$) adj3 (metatarsal$ or metatarsus or metatarsi or tarsus or tarsi or tarsal or tarsals or cuneiform or cuboid or navicular or calcane$ or talus or astragalus or tali or bimalleol$ or malleol$ or trimalleol$)).ti,ab. | 6155 |
| 14 | ((Pott$ or Cotton$) adj fractur$).ti,ab. | 6 |
| 15 | or/10-14 | 58991 |
| 16 | lower limb/ | 20085 |
| 17 | exp leg/ | 298829 |
| 18 | leg injury/ or exp ankle injury/ or exp foot injury/ or exp knee injury/ | 60340 |
| 19 | exp leg bone/ | 110410 |
| 20 | exp foot bone/ | 20813 |
| 21 | 16 or 17 or 18 or 19 or 20 | 464360 |
| 22 | (lower extremit$ or lower limb$).ti,ab. | 147136 |
| 23 | (leg or legs or thigh$ or knee or knees or shin or shins or shinbone$ or foot or midfoot or hindfoot or feet or ankle or ankles or anklebone$ or pilon or heel or heels or heelbone$ or toe or toes).ti,ab. | 566443 |
| 24 | (femur$ or femoral or tibia$ or fibula$ or patella$ or kneecap$ or knee cap$).ti,ab. | 331499 |
| 25 | (metatarsal$ or metatarsus or metatarsi or tarsus or tarsi or tarsal or tarsals or cuneiform or cuboid or navicular or calcane$ or talus or astragalus or tali or bimalleol$ or malleol$ or trimalleol$).ti,ab. | 46577 |
| 26 | or/21-25 | 1006325 |
| 27 | fracture/ | 85523 |
| 28 | 26 and 27 | 22519 |
| 29 | (fractur$ or trauma$).ti,ab. | 733605 |
| 30 | 21 and 29 | 61540 |
| 31 | fracture dislocation/ | 3884 |
| 32 | avulsion fracture/ | 2289 |
| 33 | comminuted fracture/ | 3704 |
| 34 | compression fracture/ | 6288 |
| 35 | fracture healing/ or exp fracture nonunion/ | 47518 |
| 36 | multiple fracture/ | 941 |
| 37 | open fracture/ | 6127 |
| 38 | stress fracture/ | 6670 |
| 39 | intraarticular fracture/ | 2030 |
| 40 | periprosthetic fracture/ | 3094 |
| 41 | joint fracture/ | 551 |
| 42 | limb fracture/ | 1835 |
| 43 | crush trauma/ | 3491 |
| 44 | (crush$ adj2 (injur$ or trauma$ or fractur$)).ti,ab. | 4724 |
| 45 | or/31-44 | 83329 |
| 46 | 26 and 45 | 34072 |
| 47 | multiple trauma/ | 15013 |
| 48 | (polytrauma$ or poly-trauma$ or multitrauma$ or multi-trauma$).ti,ab. | 7039 |
| 49 | ((complex$ or complicat$ or severe$ or severity or serious or major or multiple) adj2 (fractur$ or trauma$)).ti,ab. | 53380 |
| 50 | or/47-49 | 66224 |
| 51 | 26 and 50 | 10450 |
| 52 | 9 or 15 or 28 or 30 or 46 or 51 | 131185 |
| 53 | ((bone$ or fractur$) adj3 (heal or heals or healed or healing)).ti,ab. | 28002 |
| 54 | ((bone$ or fractur$) adj3 (union$ or nonunion$ or non union$ or ununite$)).ti,ab. | 10127 |
| 55 | ((bone$ or fractur$) adj3 (malunion$ or mal union$ or deform$)).ti,ab. | 6307 |
| 56 | (osteomyelitis or ((bone$ or fractur$) adj3 infect$)).ti,ab. | 35949 |
| 57 | exp osteomyelitis/ | 40281 |
| 58 | 53 or 54 or 55 or 56 or 57 | 93885 |
| 59 | 26 and 58 | 29521 |
| 60 | ((femur$ or femoral or tibia$ or fibula$ or patella$ or kneecap$ or knee cap$) adj3 (heal or heals or healed or healing)).ti,ab. | 1796 |
| 61 | ((metatarsal$ or metatarsus or metatarsi or tarsus or tarsi or tarsal or tarsals or cuneiform or cuboid or navicular or calcane$ or talus or astragalus or tali or bimalleol$ or malleol$ or trimalleol$) adj3 (heal or heals or healed or healing)).ti,ab. | 143 |
| 62 | ((femur$ or femoral or tibia$ or fibula$ or patella$ or kneecap$ or knee cap$) adj3 (union$ or nonunion$ or non union$ or ununite$)).ti,ab. | 2092 |
| 63 | ((metatarsal$ or metatarsus or metatarsi or tarsus or tarsi or tarsal or tarsals or cuneiform or cuboid or navicular or calcane$ or talus or astragalus or tali or bimalleol$ or malleol$ or trimalleol$) adj3 (union$ or nonunion$ or non union$ or ununite$)).ti,ab. | 183 |
| 64 | ((femur$ or femoral or tibia$ or fibula$ or patella$ or kneecap$ or knee cap$) adj3 (malunion$ or mal union$ or deform$)).ti,ab. | 2283 |
| 65 | ((metatarsal$ or metatarsus or metatarsi or tarsus or tarsi or tarsal or tarsals or cuneiform or cuboid or navicular or calcane$ or talus or astragalus or tali or bimalleol$ or malleol$ or trimalleol$) adj3 (malunion$ or mal union$ or deform$)).ti,ab. | 671 |
| 66 | ((femur$ or femoral or tibia$ or fibula$ or patella$ or kneecap$ or knee cap$) adj3 (osteomyelitis or infect$)).ti,ab. | 2076 |
| 67 | ((metatarsal$ or metatarsus or metatarsi or tarsus or tarsi or tarsal or tarsals or cuneiform or cuboid or navicular or calcane$ or talus or astragalus or tali or bimalleol$ or malleol$ or trimalleol$) adj3 (osteomyelitis or infect$)).ti,ab. | 467 |
| 68 | or/60-67 | 9196 |
| 69 | limb salvage/ | 9256 |
| 70 | ((limb or limbs) adj3 (restor$ or reconstruct$ or salvag$)).ti,ab. | 11320 |
| 71 | exp Ilizarov technique/ | 1884 |
| 72 | external fixator/ | 5920 |
| 73 | orthopedic fixation device/ | 1750 |
| 74 | fracture external fixation/ | 7588 |
| 75 | fracture fixation/ | 22698 |
| 76 | leg lengthening/ | 3097 |
| 77 | Ilizarov.ti,ab. | 2563 |
| 78 | (external adj2 (fixat$ or frame$ or cage$)).ti,ab. | 12015 |
| 79 | (circular adj2 (fixat$ or frame$ or cage$)).ti,ab. | 743 |
| 80 | Taylor Spatial Frame$.ti,ab. | 226 |
| 81 | TSF.ti,ab. | 1670 |
| 82 | True Lok Hex.ti,ab. | 0 |
| 83 | TLHex.ti,ab. | 0 |
| 84 | or/69-83 | 56536 |
| 85 | 26 and 84 | 27815 |
| 86 | 52 or 59 or 68 or 85 | 157443 |
| 87 | exp qualitative research/ | 82975 |
| 88 | exp questionnaire/ | 742286 |
| 89 | self report/ | 125667 |
| 90 | exp attitude/ | 796988 |
| 91 | ethnology/ | 74230 |
| 92 | discourse analysis.mp. | 2805 |
| 93 | content analysis.mp. | 37654 |
| 94 | ethnographic research.mp. | 1712 |
| 95 | ethnological research.mp. | 9 |
| 96 | constant comparative method.mp. | 2131 |
| 97 | qualitative validity.mp. | 218 |
| 98 | purposive sample.mp. | 4959 |
| 99 | observational method$.mp. | 2383 |
| 100 | field stud$.mp. | 21707 |
| 101 | theoretical sampl$.mp. | 995 |
| 102 | phenomenology.mp. | 18275 |
| 103 | phenomenological research.mp. | 562 |
| 104 | life experience$.mp. | 8583 |
| 105 | or/87-104 | 1669498 |
| 106 | interview$.mp. or interview/ or semi structured interview/ or structured interview/ or exp telephone interview/ or unstructured interview/ | 527241 |
| 107 | qualitative.mp. | 341887 |
| 108 | or/106-107 | 756039 |
| 109 | 105 or 108 | 2146655 |
| 110 | ethnograph$.mp. | 13483 |
| 111 | phenomenol$.mp. | 34484 |
| 112 | grounded theory.mp. | 15585 |
| 113 | (grounded adj (theor$ or study or studies or research or analys?s)).mp. | 15800 |
| 114 | (emic or etic or hermeneutic$ or heuristic$ or semiotic$).mp. | 20525 |
| 115 | (data adj1 saturat$).tw. | 1840 |
| 116 | participant observation/ | 5514 |
| 117 | participant observ$.tw. | 5276 |
| 118 | (action research or cooperative inquir$ or co operative inquir$ or co-operative inquir$).mp. | 5701 |
| 119 | (field adj (study or studies or research or observation$)).tw. | 23130 |
| 120 | theoretical sampl$.mp. | 995 |
| 121 | (purpos$ adj4 sampl$).mp. | 22243 |
| 122 | (focus adj group$).mp. | 61912 |
| 123 | (account or accounts or unstructured or open-ended or open ended or text$ or narrative$).mp. | 806418 |
| 124 | (life world or life-world or conversation analys?s or personal experience$ or theoretical saturation).mp. | 58012 |
| 125 | lived experience$.tw. | 7786 |
| 126 | (theme$ or thematic).mp. | 150616 |
| 127 | (observational adj (method$ or research or stud$)).mp. | 272803 |
| 128 | questionnaire$.mp. | 1003764 |
| 129 | content analysis/ | 16721 |
| 130 | thematic analysis.mp. | 29893 |
| 131 | discourse analys?s.mp. | 2843 |
| 132 | ((discourse$ or discurs$) adj3 analys?s).tw. | 2666 |
| 133 | (constant adj (comparative or comparison)).mp. | 6123 |
| 134 | narrative analys?s.mp. | 1595 |
| 135 | or/110-134 | 2289050 |
| 136 | 109 or 135 | 3363198 |
| 137 | 86 and 136 | 14058 |
| 138 | survey$.mp. | 1520579 |
| 139 | (mixed method$ or multimethod$ or multi-method$ or multi method$).mp. | 33511 |
| 140 | (patient$ adj5 (attitude$ or belief$ or believ$ or experienc$ or opinion$ or perceiv$ or perception$ or perspective$ or preference$ or view or views or viewpoint$)).ti,ab. | 444997 |
| 141 | or/138-140 | 1936448 |
| 142 | 141 and 86 | 7186 |
| 143 | 137 or 142 | 19569 |
| 144 | (animal/ or animal experiment/ or animal model/ or animal tissue/ or nonhuman/) not exp human/ | 6188171 |
| 145 | (rat or rats or mouse or mice or rodent or rodents or swine or porcine or murine or sheep or lamb or lambs or ewe or ewes or pig or pigs or piglet or piglets or sow or sows or rabbit or rabbits or kitten or kittens or dog or dogs or puppy or puppies or monkey or monkeys or horse or horses or foal or foals or equine or calf or calves or cattle or heifer or heifers or hamster or hamsters or chicken or chickens or livestock or goat or goats).ti. | 2281210 |
| 146 | 144 or 145 | 6620912 |
| 147 | 143 not 146 | 18926 |
| 148 | limit 147 to english language | 17053 |
| 149 | limit 148 to conference abstracts | 4177 |
| 150 | 148 not 149 | 12876 |

**PsycINFO**

via Ovid <http://ovidsp.ovid.com/>

1806 to 2020 November week 1

Search date: 13^th^ November 2020

Records retrieved: 358

| 1 | ((fractur$ or trauma$) adj3 (lower extremit$ or lower limb$)).ti,ab,id. | 85 |
| --- | --- | --- |
| 2 | ((fractur$ or trauma$) adj3 (leg or legs or thigh$ or knee or knees or shin or shins or shinbone$ or foot or midfoot or hindfoot or feet or ankle or ankles or anklebone$ or pilon or heel or heels or heelbone$ or toe or toes)).ti,ab,id. | 86 |
| 3 | ((fractur$ or trauma$) adj3 (femur$ or femoral or tibia$ or fibula$ or patella$ or kneecap$ or knee cap$)).ti,ab,id. | 226 |
| 4 | ((fractur$ or trauma$) adj3 (metatarsal$ or metatarsus or metatarsi or tarsus or tarsi or tarsal or tarsals or cuneiform or cuboid or navicular or calcane$ or talus or astragalus or tali or bimalleol$ or malleol$ or trimalleol$)).ti,ab,id. | 13 |
| 5 | ((Pott$ or Cotton$) adj fractur$).ti,ab,id. | 0 |
| 6 | or/1-5 | 404 |
| 7 | "leg (anatomy)"/ or thigh/ or ankle/ or "feet (anatomy)"/ or knee/ | 3550 |
| 8 | (lower extremit$ or lower limb$).ti,ab,id. | 5101 |
| 9 | (leg or legs or thigh$ or knee or knees or shin or shins or shinbone$ or foot or midfoot or hindfoot or feet or ankle or ankles or anklebone$ or pilon or heel or heels or heelbone$ or toe or toes).ti,ab,id. | 27555 |
| 10 | (femur$ or femoral or tibia$ or fibula$ or patella$ or kneecap$ or knee cap$).ti,ab,id. | 3476 |
| 11 | (metatarsal$ or metatarsus or metatarsi or tarsus or tarsi or tarsal or tarsals or cuneiform or cuboid or navicular or calcane$ or talus or astragalus or tali or bimalleol$ or malleol$ or trimalleol$).ti,ab,id. | 718 |
| 12 | or/7-11 | 33684 |
| 13 | Trauma/ or Injuries/ | 34722 |
| 14 | 12 and 13 | 852 |
| 15 | (fractur$ or trauma$).ti,ab,id. | 117553 |
| 16 | 7 and 15 | 105 |
| 17 | (crush$ adj2 (injur$ or trauma$ or fractur$)).ti,ab,id. | 218 |
| 18 | (polytrauma$ or poly-trauma$ or multitrauma$ or multi-trauma$).ti,ab,id. | 281 |
| 19 | ((complex$ or complicat$ or severe$ or severity or serious or major or multiple) adj2 (fractur$ or trauma$)).ti,ab,id. | 7000 |
| 20 | or/17-19 | 7462 |
| 21 | 12 and 20 | 105 |
| 22 | ((bone$ or fractur$) adj3 (heal or heals or healed or healing)).ti,ab,id. | 79 |
| 23 | ((bone$ or fractur$) adj3 (union$ or nonunion$ or non union$ or ununite$)).ti,ab,id. | 6 |
| 24 | ((bone$ or fractur$) adj3 (malunion$ or mal union$ or deform$)).ti,ab,id. | 29 |
| 25 | (osteomyelitis or ((bone$ or fractur$) adj3 infect$)).ti,ab,id. | 109 |
| 26 | or/22-25 | 218 |
| 27 | 12 and 26 | 48 |
| 28 | ((femur$ or femoral or tibia$ or fibula$ or patella$ or kneecap$ or knee cap$) adj3 (heal or heals or healed or healing)).ti,ab,id. | 2 |
| 29 | ((metatarsal$ or metatarsus or metatarsi or tarsus or tarsi or tarsal or tarsals or cuneiform or cuboid or navicular or calcane$ or talus or astragalus or tali or bimalleol$ or malleol$ or trimalleol$) adj3 (heal or heals or healed or healing)).ti,ab,id. | 0 |
| 30 | ((femur$ or femoral or tibia$ or fibula$ or patella$ or kneecap$ or knee cap$) adj3 (union$ or nonunion$ or non union$ or ununite$)).ti,ab,id. | 3 |
| 31 | ((metatarsal$ or metatarsus or metatarsi or tarsus or tarsi or tarsal or tarsals or cuneiform or cuboid or navicular or calcane$ or talus or astragalus or tali or bimalleol$ or malleol$ or trimalleol$) adj3 (union$ or nonunion$ or non union$ or ununite$)).ti,ab,id. | 0 |
| 32 | ((femur$ or femoral or tibia$ or fibula$ or patella$ or kneecap$ or knee cap$) adj3 (malunion$ or mal union$ or deform$)).ti,ab,id. | 6 |
| 33 | ((metatarsal$ or metatarsus or metatarsi or tarsus or tarsi or tarsal or tarsals or cuneiform or cuboid or navicular or calcane$ or talus or astragalus or tali or bimalleol$ or malleol$ or trimalleol$) adj3 (malunion$ or mal union$ or deform$)).ti,ab,id. | 1 |
| 34 | ((femur$ or femoral or tibia$ or fibula$ or patella$ or kneecap$ or knee cap$) adj3 (osteomyelitis or infect$)).ti,ab,id. | 1 |
| 35 | ((metatarsal$ or metatarsus or metatarsi or tarsus or tarsi or tarsal or tarsals or cuneiform or cuboid or navicular or calcane$ or talus or astragalus or tali or bimalleol$ or malleol$ or trimalleol$) adj3 (osteomyelitis or infect$)).ti,ab,id. | 0 |
| 36 | or/28-35 | 12 |
| 37 | ((limb or limbs) adj3 (restor$ or reconstruct$ or salvag$)).ti,ab,id. | 64 |
| 38 | Ilizarov.ti,ab,id. | 6 |
| 39 | (external adj2 (fixat$ or frame$ or cage$)).ti,ab,id. | 346 |
| 40 | (circular adj2 (fixat$ or frame$ or cage$)).ti,ab,id. | 38 |
| 41 | Taylor Spatial Frame$.ti,ab,id. | 0 |
| 42 | TSF.ti,ab,id. | 151 |
| 43 | True Lok Hex.ti,ab,id. | 0 |
| 44 | TLHex.ti,ab,id. | 0 |
| 45 | 37 or 38 or 39 or 40 or 41 or 42 or 43 or 44 | 600 |
| 46 | 12 and 45 | 38 |
| 47 | 6 or 14 or 16 or 21 or 27 or 36 or 46 | 1311 |
| 48 | exp qualitative methods/ | 16111 |
| 49 | qualitative measures/ | 77 |
| 50 | exp questionnaires/ | 20629 |
| 51 | exp attitudes/ | 389141 |
| 52 | discourse analysis.mp. | 12695 |
| 53 | content analysis.mp. | 28471 |
| 54 | ethnographic research.mp. | 2965 |
| 55 | ethnological research.mp. | 11 |
| 56 | constant comparative method.mp. | 1838 |
| 57 | qualitative validity.mp. | 10 |
| 58 | purposive sample.mp. | 2717 |
| 59 | observational method$.mp. | 1135 |
| 60 | field stud$.mp,md. | 27173 |
| 61 | theoretical sampl$.mp. | 589 |
| 62 | phenomenology.mp. | 23909 |
| 63 | observation methods/ | 5564 |
| 64 | phenomenological research.mp. | 1797 |
| 65 | life experience$.mp. | 35709 |
| 66 | or/48-65 | 540654 |
| 67 | interview$.mp. or interviews/ | 419463 |
| 68 | qualitative.mp. | 174914 |
| 69 | 67 or 68 | 505703 |
| 70 | 66 or 69 | 928690 |
| 71 | ethnograph$.mp. | 30018 |
| 72 | phenomenol$.mp. | 45973 |
| 73 | grounded theory.mp. | 16651 |
| 74 | (grounded adj (theor$ or study or studies or research or analys?s)).mp. | 17122 |
| 75 | (emic or etic or hermeneutic$ or heuristic$ or semiotic$).mp. | 28547 |
| 76 | (data adj1 saturat$).tw. | 355 |
| 77 | participant observ$.tw. | 9083 |
| 78 | (action research or cooperative inquir$ or co operative inquir$ or co-operative inquir$).mp. | 9671 |
| 79 | (field adj (study or studies or research or observation$)).tw. | 11972 |
| 80 | theoretical sampl$.mp. | 589 |
| 81 | (purpos$ adj4 sampl$).mp. | 10444 |
| 82 | (focus adj group$).mp. | 37495 |
| 83 | (account or accounts or unstructured or open-ended or open ended or text$ or narrative$).mp. | 364363 |
| 84 | (life world or life-world or conversation analys?s or personal experience$ or theoretical saturation).mp. | 15448 |
| 85 | lived experience$.tw. | 15376 |
| 86 | (theme$ or thematic).mp. | 139680 |
| 87 | (observational adj (method$ or research or stud$)).mp. | 12533 |
| 88 | field stud$.mp,md. | 27173 |
| 89 | questionnaire$.mp. | 421911 |
| 90 | content analysis.mp. | 28471 |
| 91 | thematic analysis.mp. | 15271 |
| 92 | discourse analys?s.mp. | 12817 |
| 93 | ((discourse$ or discurs$) adj3 analys?s).tw. | 9471 |
| 94 | (constant adj (comparative or comparison)).mp. | 4876 |
| 95 | narrative analys?s.mp. | 2768 |
| 96 | qualitative study.md. | 240446 |
| 97 | or/71-96 | 1128825 |
| 98 | 70 or 97 | 1585618 |
| 99 | 47 and 98 | 321 |
| 100 | exp surveys/ | 10546 |
| 101 | survey$.ti,ab. | 305436 |
| 102 | (mixed method$ or multimethod$ or multi-method$ or multi method$).mp. | 31860 |
| 103 | mixed methods research/ | 573 |
| 104 | (patient$ adj5 (attitude$ or belief$ or believ$ or experienc$ or opinion$ or perceiv$ or perception$ or perspective$ or preference$ or view or views or viewpoint$)).ti,ab. | 61559 |
| 105 | or/100-104 | 382477 |
| 106 | 47 and 105 | 99 |
| 107 | 99 or 106 | 373 |
| 108 | (rat or rats or mouse or mice or rodent or rodents or swine or porcine or murine or sheep or lamb or lambs or ewe or ewes or pig or pigs or piglet or piglets or sow or sows or rabbit or rabbits or kitten or kittens or dog or dogs or puppy or puppies or monkey or monkeys or horse or horses or foal or foals or equine or calf or calves or cattle or heifer or heifers or hamster or hamsters or chicken or chickens or livestock or goat or goats).ti. | 154261 |
| 109 | 107 not 108 | 370 |
| 110 | limit 109 to english language | 358 |

**CINAHL Complete**

via Ebsco <https://www.ebsco.com/>

Inception to 12^th^ November 2020

Search date: 13^TH^ November 2020

Records retrieved: 4869

S1 (MH "Ankle Fractures") 1,765

S2 (MH "Femoral Fractures") 4,929

S3 (MH "Tibial Fractures+") 3,906

S4 (MH "Knee Fractures+") 349

S5 (MH "Foot Fractures+") 1,170

S6 (MH "Metatarsal Fractures+") 336

S7 (MH "Fibula Fractures") 244

S8 S1 OR S2 OR S3 OR S4 OR S5 OR S6 OR S7 11,391

S9 TI ( (fractur* or trauma*) N3 (lower extremit* or lower limb*) ) OR AB ( (fractur* or trauma*) N3 (lower extremit* or lower limb*) ) 1,494

S10 TI ( (fractur* or trauma*) N3 (leg or legs or thigh* or knee or knees or shin or shins or shinbone* or foot or midfoot or hindfoot or feet or ankle or ankles or anklebone* or pilon or heel or heels or heelbone* or toe or toes) ) OR AB ( (fractur* or trauma*) N3 (leg or legs or thigh* or knee or knees or shin or shins or shinbone* or foot or midfoot or hindfoot or feet or ankle or ankles or anklebone* or pilon or heel or heels or heelbone* or toe or toes) ) 4,595

S11 TI ( (fractur* or trauma*) N3 (femur* or femoral or tibia* or fibula* or patella* or kneecap* or knee N1 cap*) ) OR AB ( (fractur* or trauma*) N3 (femur* or femoral or tibia* or fibula* or patella* or kneecap* or knee N1 cap*) ) 12,089

S12 TI ( (fractur* or trauma*) N3 (metatarsal* or metatarsus or metatarsi or tarsus or tarsi or tarsal or tarsals or cuneiform or cuboid or navicular or calcane* or talus or astragalus or tali or bimalleol* or malleol* or trimalleol*) ) OR AB ( (fractur* or trauma*) N3 (metatarsal* or metatarsus or metatarsi or tarsus or tarsi or tarsal or tarsals or cuneiform or cuboid or navicular or calcane* or talus or astragalus or tali or bimalleol* or malleol* or trimalleol*) ) 2,412

S13 TI ( (Pott* or Cotton*) N1 fractur* ) OR AB ( (Pott* or Cotton*) N1 fractur* ) 3

S14 S9 OR S10 OR S11 OR S12 OR S13 18,505

S15 (MH "Lower Extremity") OR (MH "Foot+") OR (MH "Knee") OR (MH "Leg") OR (MH "Thigh") 49,652

S16 (MH "Leg Injuries") OR (MH "Ankle Injuries") OR (MH "Foot Injuries") OR (MH "Toe Injuries") OR (MH "Knee Injuries") 12,356

S17 (MH "Leg Bones+") 21,699

S18 (MH "Foot Bones+") 5,627

S19 S15 OR S16 OR S17 OR S18 82,885

S20 TI ( lower N1 extremit* or lower N1 limb* ) OR AB ( lower N1 extremit* or lower N1 limb* ) 32,774

S21 TI ( leg or legs or thigh* or knee or knees or shin or shins or shinbone* or foot or midfoot or hindfoot or feet or ankle or ankles or anklebone* or pilon or heel or heels or heelbone* or toe or toes ) OR AB ( leg or legs or thigh* or knee or knees or shin or shins or shinbone* or foot or midfoot or hindfoot or feet or ankle or ankles or anklebone* or pilon or heel or heels or heelbone* or toe or toes ) 150,052

S22 TI ( femur* or femoral or tibia* or fibula* or patella* or kneecap* or knee N1 cap* ) OR AB ( femur* or femoral or tibia* or fibula* or patella* or kneecap* or knee N1 cap* ) 60,488

S23 TI ( metatarsal* or metatarsus or metatarsi or tarsus or tarsi or tarsal or tarsals or cuneiform or cuboid or navicular or calcane* or talus or astragalus or tali or bimalleol* or malleol* or trimalleol* ) OR AB ( metatarsal* or metatarsus or metatarsi or tarsus or tarsi or tarsal or tarsals or cuneiform or cuboid or navicular or calcane* or talus or astragalus or tali or bimalleol* or malleol* or trimalleol* ) 10,873

S24 S19 OR S20 OR S21 OR S22 OR S23 227,765

S25 (MH "Fractures") 19,535

S26 S24 AND S25 4,995

S27 TI ( fractur* or trauma* ) OR AB ( fractur* or trauma* ) 179,214

S28 S19 AND S27 11,133

S29 (MH "Fracture Dislocation") 71

S30 (MH "Avulsion Fractures") 248

S31 (MH "Fractures, Closed") 532

S32 (MH "Fractures, Comminuted") 833

S33 (MH "Fractures, Compression") 1019

S34 (MH "Fractures, Malunited") 662

S35 (MH "Fractures, Open") 1110

S36 (MH "Fractures, Stress") 1,751

S37 (MH "Fractures, Ununited+") 2196

S38 (MH "Periprosthetic Fractures") 243

S39 (MH "Fracture Healing") 4554

S40 (MH "Crush Injuries") 56

S41 TI ( crush* N2 (injur* or trauma* or fractur*) ) OR AB ( crush* N2 (injur* or trauma* or fractur*) ) 673

S42 S29 OR S30 OR S31 OR S32 OR S33 OR S34 OR S35 OR S36 OR S37 OR S38 OR S39 OR S40 OR S41 12,085

S43 S24 AND S42 5423

S44 (MH "Multiple Trauma") 3368

S45 TI ( polytrauma* or poly-trauma* or multitrauma* or multi-trauma* ) OR AB ( polytrauma* or poly-trauma* or multitrauma* or multi-trauma* ) 1,483

S46 TI ( (complex* or complicat* or severe* or severity or serious or major or multiple) N2 (fractur* or trauma*) ) OR AB ( (complex* or complicat* or severe* or severity or serious or major or multiple) N2 (fractur* or trauma*) ) 16,446

S47 S44 OR S45 OR S46 19,554

S48 S24 AND S47 3,202

S49 (S8 OR S14 OR S26 OR S28 OR S43 OR S48) 28,740

S50 TI ( (bone* or fractur*) N3 (heal or heals or healed or healing) ) OR AB ( (bone* or fractur*) N3 (heal or heals or healed or healing) ) 2,984

S51 TI ( (bone* or fractur*) N3 (union* or nonunion* or non union* or ununite*) ) OR AB ( (bone* or fractur*) N3 (union* or nonunion* or non union* or ununite*) ) 5429

S52 TI ( (bone* or fractur*) N3 (malunion* or mal union* or deform*) ) OR AB ( (bone* or fractur*) N3 (malunion* or mal union* or deform*) ) 1203

S53 TI ( (osteomyelitis or ((bone* or fractur*) N3 infect*)) ) OR AB ( (osteomyelitis or ((bone* or fractur*) N3 infect*)) ) 6,331

S54 (MH "Osteomyelitis") 3,883

S55 S50 OR S51 OR S52 OR S53 OR S54 15,662

S56 S24 AND S55 6,460

S57 TI ( (femur* or femoral or tibia* or fibula* or patella* or kneecap* or knee cap*) N3 (heal or heals or healed or healing) ) OR AB ( (femur* or femoral or tibia* or fibula* or patella* or kneecap* or knee cap*) N3 (heal or heals or healed or healing) ) 519

S58 TI ( (metatarsal* or metatarsus or metatarsi or tarsus or tarsi or tarsal or tarsals or cuneiform or cuboid or navicular or calcane* or talus or astragalus or tali or bimalleol* or malleol* or trimalleol*) N3 (heal or heals or healed or healing) ) OR AB ( (metatarsal* or metatarsus or metatarsi or tarsus or tarsi or tarsal or tarsals or cuneiform or cuboid or navicular or calcane* or talus or astragalus or tali or bimalleol* or malleol* or trimalleol*) N3 (heal or heals or healed or healing) ) 68

S59 TI ( (femur* or femoral or tibia* or fibula* or patella* or kneecap* or knee cap*) N3 (union* or nonunion* or non union* or ununite*) ) OR AB ( (femur* or femoral or tibia* or fibula* or patella* or kneecap* or knee cap*) N3 (union* or nonunion* or non union* or ununite*) ) 752

S60 TI ( (metatarsal* or metatarsus or metatarsi or tarsus or tarsi or tarsal or tarsals or cuneiform or cuboid or navicular or calcane* or talus or astragalus or tali or bimalleol* or malleol* or trimalleol*) N3 (union* or nonunion* or non union* or ununite*) ) OR AB ( (metatarsal* or metatarsus or metatarsi or tarsus or tarsi or tarsal or tarsals or cuneiform or cuboid or navicular or calcane* or talus or astragalus or tali or bimalleol* or malleol* or trimalleol*) N3 (union* or nonunion* or non union* or ununite*) ) 107

S61 TI ( (femur* or femoral or tibia* or fibula* or patella* or kneecap* or knee cap*) N3 (malunion* or mal union* or deform*) ) OR AB ( (femur* or femoral or tibia* or fibula* or patella* or kneecap* or knee cap*) N3 (malunion* or mal union* or deform*) ) 762

S62 TI ( (metatarsal* or metatarsus or metatarsi or tarsus or tarsi or tarsal or tarsals or cuneiform or cuboid or navicular or calcane* or talus or astragalus or tali or bimalleol* or malleol* or trimalleol*) N3 (malunion* or mal union* or deform*) ) OR AB ( (metatarsal* or metatarsus or metatarsi or tarsus or tarsi or tarsal or tarsals or cuneiform or cuboid or navicular or calcane* or talus or astragalus or tali or bimalleol* or malleol* or trimalleol*) N3 (malunion* or mal union* or deform*) ) 250

S63 TI ( (femur* or femoral or tibia* or fibula* or patella* or kneecap* or knee cap*) N3 (osteomyelitis or infect*) ) OR AB ( (femur* or femoral or tibia* or fibula* or patella* or kneecap* or knee cap*) N3 (osteomyelitis or infect*) ) 574

S64 TI ( (metatarsal* or metatarsus or metatarsi or tarsus or tarsi or tarsal or tarsals or cuneiform or cuboid or navicular or calcane* or talus or astragalus or tali or bimalleol* or malleol* or trimalleol*) N3 (osteomyelitis or infect*) ) OR AB ( (metatarsal* or metatarsus or metatarsi or tarsus or tarsi or tarsal or tarsals or cuneiform or cuboid or navicular or calcane* or talus or astragalus or tali or bimalleol* or malleol* or trimalleol*) N3 (osteomyelitis or infect*) ) 169

S65 S57 OR S58 OR S59 OR S60 OR S61 OR S62 OR S63 OR S64 2,942

S66 (MH "Limb Salvage") 1,615

S67 TI ( (limb or limbs) N3 (restor* or reconstruct* or salvag*) ) OR AB ( (limb or limbs) N3 (restor* or reconstruct* or salvag*) ) 2,123

S68 (MH "External Fixators") 689

S69 (MH "Orthopedic Fixation Devices") 16,210

S70 (MH "Fracture Fixation") 15,069

S71 (MH "Bone Lengthening") 824

S72 TI Ilizarov OR AB Ilizarov 647

S73 TI ( external N2 (fixat* or frame* or cage*) ) OR AB ( external N2 (fixat* or frame* or cage*) ) 2,839

S74 TI ( circular N2 (fixat* or frame* or cage*) ) OR AB ( circular N2 (fixat* or frame* or cage*) ) 240

S75 TI Taylor Spatial Frame* OR AB Taylor Spatial Frame* 117

S76 TI TSF OR AB TSF 257

S77 TI True Lok Hex OR AB True Lok Hex 0

S78 TI TLHex OR AB TLHex 0

S79 S66 OR S67 OR S68 OR S69 OR S70 OR S71 OR S72 OR S73 OR S74 OR S75 OR S76 OR S77 OR S78 28,970

S80 S24 AND S79 13,643

S81 S49 OR S56 OR S65 OR S80 36,855

S82 (MH "Qualitative Studies+") 151,946

S83 (MH "Qualitative Validity+") 1,665

S84 (MH "Questionnaires+") 421,021

S85 (MH "Attitude+") 458.184

S86 (MH "Focus Groups") 44,394

S87 (MH "Discourse Analysis") 4,956

S88 (MH "Content Analysis") 36,541

S89 (MH "Constant Comparative Method") 7,355

S90 (MH "Purposive Sample") 32,041

S91 (MH "Observational Methods+") 21,702

S92 (MH "Field Studies") 3,339

S93 (MH "Theoretical Sample") 1,774

S94 (MH "Phenomenology") 3,681

S95 (MH "Life Experiences+") 42,751

S96 S82 OR S83 OR S84 OR S85 OR S86 OR S87 OR S88 OR S89 OR S90 OR S91 OR S92 OR S93 OR S94 OR S95 908,869

S97 TX interview* OR TX interview* 502,097

S98 (MH "Interviews+") 224,279

S99 TX qualitative OR TX qualitative 279,745

S100 S97 OR S98 OR S99 623,690

S101 S96 OR S100 1,230,261

S102 TI ((discourse* or discurs*) N3 analys*) OR AB ((discourse* or discurs*) N3 analys*) 2,762

S103 TI content N1 analys* OR AB content N1 analys* 26,148

S104 TI ethnograph* OR AB ethnograph* 9702

S105 TI ethnolog* OR AB ethnolog* 62

S106 TI constant N1 (comparative OR comparison*) OR AB constant N1 (comparative OR comparison*) 4,682

S107 TI "purposive sample" OR AB "purposive sample" 3,513

S108 TI purpos* N4 sampl* OR AB purpos* N4 sampl* 9,267

S109 TI ( field N1 (study or studies or research or observation*) ) OR AB ( field N1 (study or studies or research or observation*) ) 6962

S110 TI theoretical N1 sampl* OR AB theoretical N1 sampl* 842

S111 TI phenomenol* OR AB phenomenol* 17,135

S112 TI ( (life or lived) N1 experience* ) OR AB ( (life or lived) N1 experience* ) 13,592

S113 (MH "Grounded Theory") 16,154

S114 TI ( (grounded N1 (theor* or study or studies or research or analys*) ) OR AB ( (grounded N1 (theor* or study or studies or research or analys*) ) 12,777

S115 TI ( emic or etic or hermeneutic* or heuristic* or semiotic* ) OR AB ( emic or etic or hermeneutic* or heuristic* or semiotic* ) 7,300

S116 TI data N1 saturat* OR AB data N1 saturat* 955

S117 TI participant observ* OR AB participant observ* 11,279

S118 TI ( "action research" or cooperative inquir* or co operative inquir* or co-operative inquir* ) OR AB ( "action research" or cooperative inquir* or co operative inquir* or co-operative inquir* ) 4,303

S119 TI focus N1 group* OR AB focus N1 group* 36,156

S120 TI ( account or accounts or unstructured or open N1 ended or text* or narrative* ) OR AB ( account or accounts or unstructured or open N1 ended or text* or narrative* ) OR (MH "Narratives") 168,242

S121 TI ( life N1 world or conversation N1 analys* or personal N1 experience* or "theoretical saturation" ) OR AB ( life N1 world or conversation N1 analys* or personal N1 experience* or "theoretical saturation" ) 7749

S122 TI ( theme* or thematic* ) OR AB ( theme* or thematic* ) 88,322

S123 (MH "Thematic Analysis") 65,560

S124 TI ( observational N1 (method* or research or stud*) ) OR AB ( observational N1 (method* or research or stud*) ) 55,761

S125 TI questionnaire* OR AB questionnaire* 223,096

S126 TI narrative N1 analys* OR AB narrative N1 analys* 2,033

S127 S102 OR S103 OR S104 OR S105 OR S106 OR S107 OR S108 OR S109 OR S110 OR S111 OR S112 OR S113 OR S114 OR S115 OR S116 OR S117 OR S118 OR S119 OR S120 OR S121 OR S122 OR S123 OR S124 OR S125 OR S126 595,622

S128 S101 OR S127 1,453,969

S129 (MH "Surveys") or (MH "Survey Research") 164,827

S130 TI survey* OR AB survey* 273,768

S131 (MH "Multimethod Studies") 15,619

S132 TI ( (mixed N1 method* or multimethod* or multi N1method*) ) OR AB ( (mixed N1 method* or multimethod* or multi N1 method*) ) 20,616

S133 TI ( patient* N5 (attitude* or belief* or believ* or experienc* or opinion* or perceiv* or perception* or perspective* or preference* or view or views or viewpoint*) ) OR AB ( patient* N5 (attitude* or belief* or believ* or experienc* or opinion* or perceiv* or perception* or perspective* or preference* or view or views or viewpoint*) ) 122,993

S134 S129 OR S130 OR S131 OR S132 OR S133 467,300

S135 S128 OR S134 1,636,669

S136 S81 AND S135 4,969

S137 TI (rat or rats or mouse or mice or rodent or rodents or swine or porcine or murine or sheep or lamb or lambs or ewe or ewes or pig or pigs or piglet or piglets or sow or sows or rabbit or rabbits or kitten or kittens or dog or dogs or puppy or puppies or monkey or monkeys or horse or horses or foal or foals or equine or calf or calves or cattle or heifer or heifers or hamster or hamsters or chicken or chickens or livestock or goat or goats) 101,722

S138 S136 not S137 4,825

S139 S136 not S137 Limiters - English Language 4,869
