# Supplementary material for: Exploring what is important to patients with regards to quality of life after experiencing a lower limb reconstructive procedure: a qualitative evidence synthesis
Source: Health Qual Life Outcomes. 2021 May 31;19:158. doi: 10.1186/s12955-021-01795-9 (PMC8166062; doi:10.1186/s12955-021-01795-9)
Supplement: Supplementary file 2 — Additional file 2. Appendix 2: Quality assessment outcome table. [file 12955_2021_1795_MOESM2_ESM.docx]

Appendix 2. CASP of the included studies.

| **Study** | **A1: Was there a clear statement of the aims of the research?** | **A2: Is a qualitative methodology appropriate?** | **A3: Was the research design appropriate to address the aims of the research?** | **A4: Was the recruitment strategy appropriate to the aims of the research?** | **A5: Was the data collected in a way that addressed the research issue?** | **A6: Has the relationship between researcher and participants been adequately considered?** | **B7: Have ethical issues been taken into consideration?** | **B8: Was the data analysis sufficiently rigorous?** | **B9: Is there a clear statement of the findings?** | **C10: How valuable is the research?** |
| --- | --- | --- | --- | --- | --- | --- | --- | --- | --- | --- |
| Aravind | Yes | Yes | Yes | Yes | Yes | Can’t tell | Can’t tell | Yes | Yes | Valuable |
| Bernhoff | Yes | Yes | Yes | Yes | Can't tell | No | Yes | Can’t tell | Yes | Valuable |
| Griffiths | Yes | Yes | Yes | Yes | Yes | No | Can’t tell | Yes | Yes | Valuable |
| McPhail | Yes | Yes | Yes | Yes | Yes | Yes | No | Yes | Yes | Very valuable |
| Mundy | Yes | Yes | Yes | Yes | Can’t tell | No | Can’t tell | Yes | Can’t tell | Valuable |
| Phelps | Yes | Yes | Can’t tell | Can’t tell | Yes | Can’t tell | Can’t tell | Yes | Yes | Valuable |
| Rees | Yes | Yes | Yes | Can’t tell | Yes | Yes | Yes | Yes | Yes | Very valuable |
| Trickett | Yes | Yes | Yes | Yes | Yes | Can’t tell | Can’t tell | Can’t tell | Yes | Unsure |
| Tutton | Yes | Yes | Yes | Yes | Yes | Can’t tell | Can’t tell | Can’t tell | Can’t tell | Unsure |
